# In Vitro Study on the Regulation of Annexin IV and VEGF by hCG in the Human Endometrium

**DOI:** 10.1155/2020/8892930

**Published:** 2020-10-23

**Authors:** Shaoyuan Xu, Jie Li, Xiaoyan Chen, Beiyu Liu

**Affiliations:** ^1^Reproductive Medicine Center, Renmin Hospital, Hubei University of Medicine, Shiyan, Hubei 442000, China; ^2^Human Reproductive Center of the First Affiliated Hospital of Sun Yat-Sen University, Guangzhou 510080, China; ^3^Human Reproductive Center of Shanghai Tenth People's Hospital, Shanghai 200000, China

## Abstract

**Objective:**

Whether changes in vascular endothelial growth factor (VEGF) and annexin IV during implantation are regulated through the LH/hCG-R needs further research. To investigate the mechanism of hCG on the expression of annexin IV and VEGF in human endometrial cells.

**Methods:**

Endometrial cells were isolated and identified from human specimens. The proportion of glandular and epithelial cells was analyzed. Annexin IV and VEGF were analyzed by qRT-PCR (mRNA), western blot (proteins), and immunohistochemistry (proteins). Protein location was identified by immunohistochemistry. The cells were cultured with hCG, hCG/PD98059 (a MAPK inhibitor), or no treatment (control).

**Results:**

The proportions between the glandular epithelial cells and stromal cells at inoculation and when adding hCG were 25.8 ± 0.2% and 27.8 ± 0.04%, respectively (*P* > 0.05). LH/hCG-R, annexin IV, and VEGF were found in the cytoplasm of endometrial cells. After 2, 6, 12, and 24 h of hCG treatment, compared with 1 h, VEGF mRNA was increased by 1.25-fold, 3.19-fold, 4.21-fold, and 4.86-fold and annexin IV by 2.23-fold, 3.37-fold, 5.14-fold, and 5.02-fold. Compared with the control group, annexin IV mRNA and protein were increased in the hCG and hCG/PD98059 groups (mRNA/protein: 1.99-fold/1.80-fold and 2.33-fold/1.93-fold, *P* < 0.05). Compared with the control group, VEGF mRNA and protein were increased in the hCG group (mRNA/protein: 2.30-fold/1.86-fold), but not in the hCG/PD98059 group.

**Conclusion:**

hCG could upregulate the mRNA and protein expression of annexin IV and VEGF. The upregulation of annexin IV by hCG could not be inhibited by PD98059, but the upregulation of VEGF by hCG could.

## 1. Introduction

The actions of luteinizing hormone (LH) and human chorionic gonadotropin (hCG) are mediated by the same receptor (LH/hCG-R) in the human sex gland cells [1]. LH and hCG combine with the LH/hCG-R to activate the G protein-coupled Ac (adenylate cyclase)-cAMP (cyclic adenosine monophosphate) signaling pathway [1]. LH/hCG-R was found in humans and other several species (including pig, free, cat, cattle, and monkey) [1]. The protein and mRNA levels of the LH/hCG-R reach their maximum expression during the implantation window [2–4].

Under the action of serum LH peak or exogenous hCG, the endometrial tissue transforms from the proliferative phase to the secretory phase, entering the implantation window. During this process, many local molecules such as integrin, adhesion molecules, leukemia inhibitory factor (LIF), vascular endothelial growth factor (VEGF), and epidermal growth factor are also expressed [5–7]. These molecules regulate the mutual recognition and receptivity between the endometrium and the embryo before implantation, which is essential for successful implantation.

Annexin IV is a protein playing a role in cell fusion, cytomembrane throughput, and cytoskeletal activities [8]. VEGF regulates angiogenesis and vascular permeability [9]. These two proteins play key roles in embryo implantation and viability [10, 11]. Nevertheless, whether the changes in the expression levels of these genes and proteins during the implantation are directly or indirectly regulated through the binding of LH and hCG to the LH/hCG-R needs further research.

Therefore, the aim of the present study was to investigate the mechanism of hCG on the expression of annexin IV and VEGF in human endometrial cells in an in vitro culture model.

## 2. Materials and Methods

### 2.1. Tissue Collection

This study enrolled patients scheduled to undergo hysteroscopic endometrial curettage or surgical uterus resection due to uterine fibroids in the late or middle proliferation phase of the menstrual cycle. The inclusion criteria were as follows: (1) 20–45 years of age, (2) regular menstrual cycle, and (3) no history of taking hormone drugs in the past 3 months. Endometrial tissues were collected from seven women by hysteroscopy (*n* = 5) or hysterectomy (*n* = 2) at the First Affiliated Hospital of Sun Yat-Sen University between March 2012 and December 2012. The study was approved by the Ethics Committee of the First Affiliated Hospital of Sun Yat-Sen University (2012-No.284). A written informed consent form was signed by all patients.

### 2.2. Primary Cell Culture and Grouping

Each endometrial sample was washed three times with sterile phosphate-buffered saline (PBS), cut into 1–2 mm [3] pieces, and digested for 40–60 min with 0.25% collagenase type I (Gibco, Invitrogen Inc., Carlsbad, CA, USA) and 0.005% DNA enzyme (Sigma, St. Louis, MO, USA). The suspension was filtered through a 150 *µ*m nylon mesh to harvest the endometrial cells. The cells were resuspended into centrifuge tubes and centrifuged for 5 min at 500 ×g. The cell pellet was collected, and the cells were maintained in a Dulbecco's modified medium (DMEM)/F12 (Gibco) supplemented with 10% fetal calf serum (Invitrogen Inc., Carlsbad, CA, USA) and 1% penicillin-streptomycin (Life Technologies Co., Grand Island, NY, USA) and under a 5% CO_2_ atmosphere at 37°C. The trypan blue method was used to test cell viability. The cells were cultured for at least 5 days prior to any experiment. The cells were plated at 5 × 10^5^ cells/plate and were cultured for 24 h. The cells were grouped as the control group, the hCG group, and the hCG/PD98059 group. The hCG group was treated with 20 U/ml hCG (Livzon Group Livzon Pharmaceutical Factory, Beijing, China) for 24 h. The hCG/PD98059 group was treated with 20 U/ml hCG and 50 *µ*mol/ml PD98059 (a selective MAPK inhibitor) for 24 h (VEGF experiments) or 12 h (annexin IV experiments). The control group was incubated without hCG or PD98059.

### 2.3. Immunocytochemistry (ICC) Detection

The cultured endometrial cells were inoculated on sterile coverslips at 1 × 10^5^/ml and cultured in an atmosphere of 5% CO_2_ at 37°C for 5 days, washed three times with PBS, soaked in methanol at −20°C for 10 min, and blocked with 5% normal goat serum (NGS) at 37°C for 30 min. The cells were incubated overnight at 4°C with a primary antibody: keratin (ab51054; 1 : 100; Abcam, Cambridge, United Kingdom) for glandular epithelial cells, vimentin (ab254015; 1 : 100; Abcam) for stromal cells, anti-LHR (sc-25828; 1 : 50; Santa Cruz Biotechnology, Santa Cruz, CA, USA), annexin IV (3175–1; 1 : 500; RabMabs, Burlingame, CA, USA), and VEGF (sc-705; 1 : 50; Santa Cruz). PBS, instead of the primary antibody, was used for negative control. The secondary antibody was HRP-linked anti-mouse IgG (7076S; 1 : 1500; Cell Signaling Technology, Inc., Danvers, MA, USA) for 30 min at room temperature. The staining was revealed with DAB for 3–5 min. The cells were counterstained with hematoxylin for 30 s. The cells were dehydrated and mounted with a mounting medium (Wuhan Boshide Biotechnology Co., Wuhan, China). Examination and photography were performed using an inverted microscope (Carl Zeiss GmbH, Oberkochen, Germany).

### 2.4. Glandular Epithelial and Stromal Cell Identification

The cells were assessed when they were inoculated and after treatment with hCG. Immunohistochemistry was used to identify the stromal cells and glandular epithelial cells in 10 fields of view under the microscope. The stromal cells and glandular epithelial cells were counted, and their proportion was calculated.

### 2.5. Quantitative-Polymerase Chain Reaction (qRT-PCR)

Total RNA was extracted using TRIzol (Invitrogen). Total RNA (2 *µ*g) was reverse-transcribed according to the manufacturer's instructions. The relative quantification of gene expression was carried out in triplicate for each gene using the 7500 Real-Time PCR System (Applied Biosystems, Foster City, CA, USA). Oligonucleotide primers were designed by Shanghai Yingjun Biotechnology Co., Ltd. (Shanghai, China). The annexin IV forward primer (GAG CAC CAT CGG CAG GGA CT), the annexin IV reverse primer (TCA TAC AGC ACC GTG GGC GT), the VEGF forward primer (ACC TCC ACC ATG CCA AGT GGT C), the VEGF reverse primer (TGT CCA CCA GGG TCT CGA TTG GA), the *β*-actin forward primer (CGG ATG TCC ACG TCA CAC TT), and the *β*-actin reverse primer (GTT GCT ATC CAG GCT GTG GT) were used for the PCR using the SYBR Green I Kit (Roche Diagnostics, Basel, Switzerland), according to the manufacturer's instructions.

### 2.6. Western Blotting

Total proteins were extracted using the RIPA buffer. Cell extracts were centrifuged, and supernatant proteins were quantified using the BCA protein assay kit (P0010; Beyotime Institute of Biotechnology, Haimen, China), according to the manufacturer's instructions. Proteins (40 *µ*g) were resolved using 10% SDS-PAGE, transferred to nitrocellulose membranes, blocked with 5% nonfat milk in tris-buffered saline (TBS) (Bio-Rad, Hercules, CA, USA), and probed separately with annexin IV antibody (annexin IV (3175–1; 1 : 5000; RabMabs) and VEGF antibody (sc-705; 1 : 200; Santa Cruz) overnight at 4°C. The membranes were washed in TBS and incubated for 1 h with anti-mouse IgG HRP-linked antibody (#7076S; 1 : 1500; Cell Signaling Technology). The membranes were exposed to autoradiographic films. Densitometric analysis of band intensity was performed using Gel Pro Analyzer 4.

### 2.7. Statistical Analysis

Data were expressed as mean ± standard deviation. Statistical analysis was performed using GraphPad Prism (GraphPad Software Inc., San Diego, CA, USA). The qRT-PCR and western blot data were analyzed by ANOVA or the Wilcoxon test. *P* values <0.05 were considered statistically significant.

## 3. Results

### 3.1. Immunocytochemistry for Glandular Epithelial and Stromal Cells

**Figure 1:**
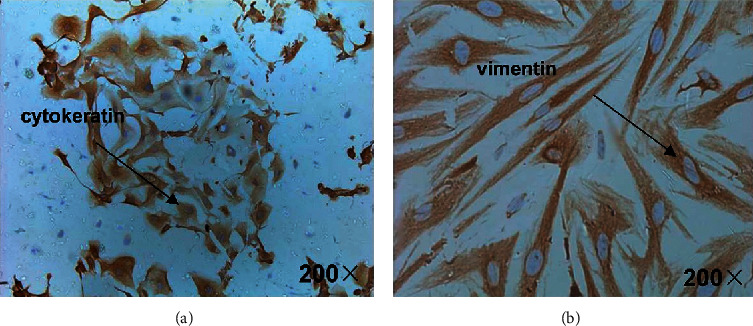
Immunocytochemistry for glandular epithelial and stromal cells. Cells isolated from human endometrium specimens were stained for cytokeratin (a) and vimentin (b). Endometrial epithelial cells stained positive for cytokeratin, while stromal cells stained positive for vimentin (magnification: 200×).

Endometrial epithelial cells stained positive for cytokeratin, while stromal cells stained positive for vimentin (Figure 1). After counting the cells and calculating their proportions, the results showed that the proportions between the glandular epithelial cells and stromal cells at inoculation and when adding hCG were 25.8 ± 0.15% and 27.8 ± 0.04%, respectively (*P* > 0.05).

### 3.2. Immunohistochemistry for LH/hCG-R

**Figure 2:**
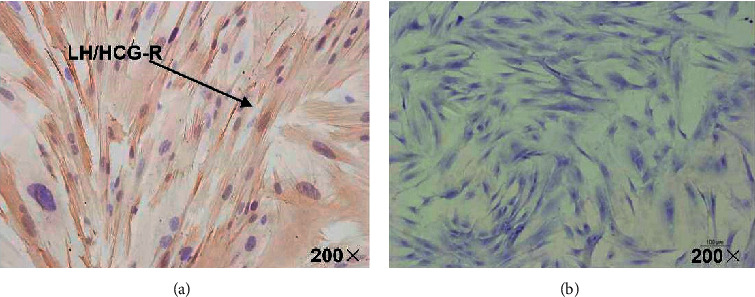
Immunohistochemistry for LH/hCG-R. LH/hCG-R was positive in the endometrial cell cytoplasm (a). LH/hCG-R was negative in control cells (b) (magnification: 200×).

Immunohistochemistry for LH/hCG-R showed that LH/hCG-R was positive in the endometrial cell cytoplasm (Figure 2(a)). LH/hCG-R was negative in control (Figure 2(b)).

### 3.3. Annexin IV and VEGF Localization

**Figure 3:**
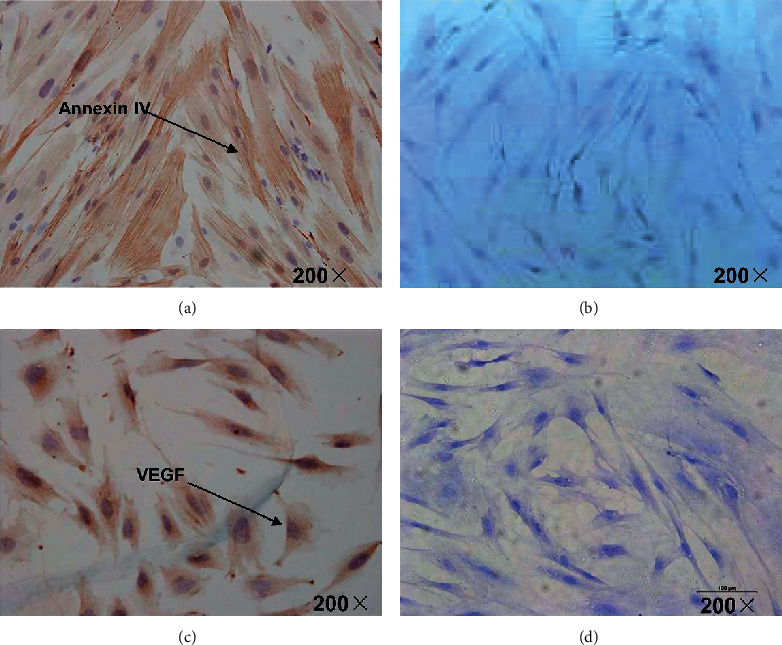
Annexin IV and VEGF localization. Immunohistochemistry for annexin IV cellular localization showed that annexin IV was positive in the endometrial cell cytoplasm (a), but not in control cells (b). VEGF was positive in the endometrial cell cytoplasm (c), but not in control cells (d) (magnification: 200×).

Immunohistochemistry for annexin IV cellular localization showed that annexin IV was positive in the endometrial cell cytoplasm (Figure 3(a)). VEGF was positive in the endometrial cell cytoplasm (Figure 3(c)). The control cells were negative (Figures 3(b) and 3(d)).

### 3.4. Expression of Annexin IV and VEGF after Treatment with hCG

**Figure 4:**
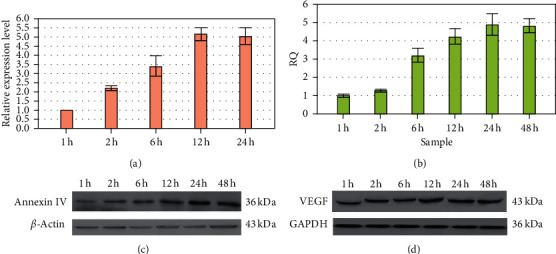
Protein expression of annexin IV and VEGF after treatment with hCG. The cells in the hCG group were treated with a final concentration of 20 IU/ml hCG. Total RNA and protein were extracted at 1, 2, 6, 12, 24, and 48 h. Annexin IV (a) and VEGF (b) protein expression levels were detected.

The cells in the hCG group were treated with a final concentration of 20 IU/ml hCG. Total RNA and protein were extracted at 1, 2, 6, 12, 24, and 48 h. Compared with 1 h of treatment, the mRNA expression of VEGF was increased by 1.25-fold at 2 h, 3.19-fold at 6 h, 4.21-fold at 12 h, 4.86-fold at 24 h, and 4.82-fold at 48 h (Figure 4(a) and Table 1). Compared with 1 h of treatment, the mRNA expression of annexin IV was increased by 2.23-fold at 2 h, 3.37-fold at 6 h, 5.14-fold at 12 h, and 5.02-fold at 24 h (Figure 4(b) and Table 2). Compared with 1 h of treatment, the protein expression of VEGF increased in time and peaked at 24 h (Figure 4(c) and Table 3). Compared with 1 h of treatment, the protein expression of annexin IV increased in time and peaked at 24 h (Figure 4(d) and Table 4).

### 3.5. Annexin IV and VEGF Expression in the Control, hCG, and hCG/PD98059 Groups

**Figure 5:**
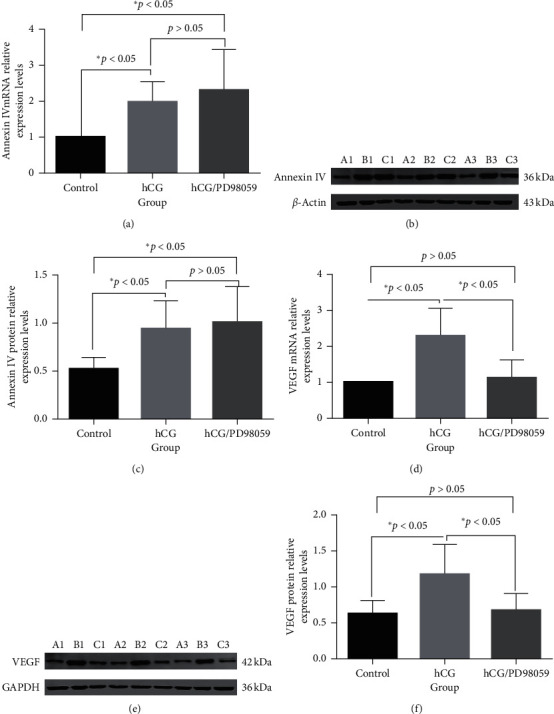
Annexin IV and VEGF expression in the control, hCG, and hCG/PD98059 groups. The cells in the hCG group were treated with a final concentration of 20 IU/ml hCG for 12 h. The cells in the hCG/PD98059 group were treated with 20 IU/ml hCG and 50 mIU/ml PD98059. mRNA levels were detected by qRT-PCR, and protein levels were detected by western blot. (a) mRNA expression of annexin IV. (b) mRNA expression of VEGF. (c) Protein expression of annexin IV. (d) Protein expression of VEGF (^*∗*^*P* < 0.05).

Compared with the control group, the annexin IV mRNA expression levels of the hCG group were significantly higher after treatment with 20 IU/ml hCG for 12h (+98%, *P* < 0.05). When treated with 20 IU/ml hCG for 12 h, the annexin IV mRNA expression levels were increased by 1.99-fold (*P* < 0.05); when treated with 20 IU/ml hCG and the MAPK inhibitor PD98059 (50 mIU/ml) for 12 h, the annexin IV mRNA expression levels were increased by 2.33-fold (*P* < 0.05) (Figure 5(a) and Table 5). There were no differences between the hCG and hCG/PD98059 groups. When treated with 20 IU/ml hCG for 12 h, VEGF mRNA expression levels were increased by 2.30-fold (*P* < 0.05); when treated with 20 IU/ml hCG and the MAPK inhibitor PD98059 (50 mIU/ml) for 12 h, VEGF mRNA expression levels were increased by 1.13-fold (*P* < 0.05) (Figure 5(b) and Table 6).

Similar results were observed for annexin IV protein expression, with 1.80-fold in the hCG group and 1.93-fold in the hCG/PD98059 group (both *P* < 0.05) (Figure 5(c) and Table 7). There were no differences between the hCG and hCG/PD98059 groups. Similar results were also observed for VEGF protein expression, with 1.86-fold in the hCG group and 1.07-fold in the hCG/PD98059 group (both *P* < 0.05) (Figure 5(d) and Table 8). There were no differences between the hCG and hCG/PD98059 groups.

## 4. Discussion

There is a possibility that VEGF and annexin IV are regulated through the LH/hCG-R during embryo implantation, but data are needed. Therefore, this study aimed to investigate the mechanism of hCG on the expression of annexin IV and VEGF in human endometrial cells. The present study suggests that hCG upregulates the mRNA and protein expression of annexin IV and VEGF. The upregulation of annexin IV by hCG did not involve the MAPK pathway, but the upregulation of VEGF by hCG did involve MAPK. These results highlight the regulation of annexin IV and VEGF during the implantation window.

Lei et al. [12] reported the expression of LH/hCG-R mRNA on human oviduct epithelial cells. Since then, many studies successively reported that there was also LH/hCG-R expression on the gamete, early embryo/blastocyst, fallopian tubes, uterus, cervix, placenta, fetal membranes, umbilical cord, and endometrial carcinoma RL95-2 cells [13–15]. The present study confirmed that LH/hCG-R protein was expressed on human endometrial glandular epithelial cells and stromal cells in vitro by immunohistochemistry.

The morphology and function of the endometrium are highly dependent on their regulation by hormones. The transformation of the proliferative phase of the endometrium to the implantation window is regulated by estrogens, progesterone, and possibly by other unknown factors. The LH peak is the most critical factor to promote oocyte maturation, embryonic development, and synchronous endometrial receptivity [16, 17]. The expression of LH/hCG-R on the endometrium prompted that LH/hCG-R may serve as a signal that prompts embryo implantation and development. On the other hand, LH/hCG-R might be involved in the dialogue between the embryo and maternal endometrium during the implantation process, and the synchronization of the two entities is worthy of further study.

The embryo in the uterine cavity and endometrium secrete proteins and local factors to achieve mutual recognition and fusion and complete the implantation process. LH and hCG combine with the LH/hCG-R and activate the G protein. The G protein further activates three downstream signaling pathways: (1) the Ac-cAMP signaling pathway; (2) the phospholipase c (PLc)-inositol triphosphate (IP3)/diacylglycerol (DAG)-PKc pathway; and (3) the MAPK pathway [18, 19].

The MAPK pathway plays an important role in cell growth, proliferation, and differentiation. The MAPK pathway mainly mediates the processes of transducing extracellular signals, and it can adjust gene expression levels and activate enzyme substrates in the cytoplasm. The MAPK pathway is involved in the regulation of steroid hormone production in human ovarian granulosa cells and placental cells [20, 21]. LH and hCG could activate the ERK1/ERK2 (the two main MAPKs) pathway of luteinized granule cells [22, 23]. The MAPK inhibitors PD98095 and U0126 can inhibit the ERK1/ERK2 pathway, thereby affecting the expression of StAR. In this study, after adding hCG and PD9809, the protein and gene expression levels of annexin IV did not show significant changes when compared with the hCG group, suggesting that the regulation of annexin IV by hCG in endometrial cells was not through the MAPK pathway. On the other hand, after treatment with hCG and PD9809, protein and gene expression levels of VEGF were downregulated compared with the hCG group, indicating the involvement of the MAPK pathway.

Annexin IV belongs to the Ca^2+^-dependent phospholipid-binding protein family. After being activated by Ca^2+^, annexin IV combines with membrane phospholipids and participate in anticoagulation, inhibition of phospholipase A_2_ activity, interactions of membrane and cytoskeleton, formation of ion channels, Ca^2+^-dependent signaling, and endocytosis and exocytosis [24]. Annexin IV is also involved in cell growth, differentiation, morphological conversion process, and anti-inflammatory properties [24, 25]. The role of annexin IV in the endometrium remains uncertain. Mirkin et al. [26] pointed out that in the menstrual cycle, annexin IV mRNA levels in the secretory phase were significantly higher than those in the proliferative phase. Ponnampalam and Rogers [27] reported annexin IV cyclical changed throughout the menstrual cycle. Their results showed that annexin IV mRNA and protein expression levels begin to increase in the early secretory phase and to peak in the midsecretory phase. The expression levels of annexin IV in the secretory phase are related to the progesterone levels [28]. In this study, protein and gene expression levels of annexin IV were significantly upregulated in the hCG group. This was different from the study by Yu and Li [14] in the RL95-2. The difference between the two studies might be due to the different concentrations of hCG intervention, the treatment time, and differences between cell lines and cells isolated from specimens.

VEGF and its receptor regulate vascular permeability [29]. Meanwhile, changes in vascular permeability are involved in the maturation of the endometrium and the implantation window [30, 31]. Hosper et al. [32] showed that VEGF presents regular periodic changes on the uterus during the menstrual cycle. Licht et al. [33, 34] introduced hCG into the uterine cavity using a microdialysis device and showed that hCG could upregulate the VEGF levels. Berndt et al. [35] confirmed the regulation of hCG on VEGF through the LH/hCG-R, so as to activate the angiogenic activity of endometrial epithelial and endothelial cells. This suggests the regulatory role of hCG on endometrial blood vessels through LH/hCG-R. Fogle et al. [36] showed that VEGF decreased with age and could be involved in infertility. Paiva et al. [37] used hCG increased VEGF expression. In the present study, the protein and mRNA expression levels of VEGF were significantly upregulated in the hCG group compared with the control group. This was supported by the literature above.

In conclusion, endometrial morphology and function transformation are regulated not only by steroid hormones, cytokines, and adhesion molecules but also by hCG or LH directly. hCG could upregulate the mRNA and protein expression levels of annexin IV and VEGF. The effect of hCG on annexin IV does not involve MAPK pathway but on VEGF involves MAPK pathway. The changes in these two proteins participate in endometrial receptivity.

## Figures and Tables

**Table 1:** VEGF mRNA relative expression levels of different hCG acting time points.

Different hCG acting time (h)	1	2	6	12	24	48
VEGF mRNA relative expression levels	1.0000	1.2549	3.1920	4.2119	4.8646	4.8219

**Table 2:** VEGF protein relative expression levels of different hCG acting time points.

Different hCG acting time (h)	1	2	6	12	24	48
VEGF protein relative expression levels	0.66828	0.674149	0.676553	0.697746	0.801305	0.772691

**Table 3:** Annexin IV mRNA relative expression levels of different hCG acting time points.

Different hCG acting time (h)	1	2	6	12	24
Annexin IV mRNA relative expression levels	1.0000	2.2341	3.3699	5.1420	5.0188

**Table 4:** Annexin IV protein relative expression levels of different hCG acting time points.

Different hCG acting time (h)	1	2	6	12	24	48
Annexin IV protein relative expression levels	0.897632	0.899066	0.932229	0.959593	0.955211	0.942279

**Table 5:** Annexin IV mRNA relative expression levels after hCG/PD98059 treating.

Sample no.	Control group	hCG group	hCG/PD98059
1	1	1.3939	1.7864
2	1	2.7089	4.0149
3	1	1.7125	1.6955
4	1	2.1326	1.8093
Mean	1	1.987 ± 0.568	2.326 ± 1.126

hCG group vs. control group *P* < 0.05; hCG/PD98059 group vs. control group *P* < 0.05; hCG/PD98059 group vs. hCG group *P* > 0.05.

**Table 6:** Annexin IV protein relative expression levels after hCG/PD98059 treating.

Sample no.	Control group	hCG group	hCG/PD98059
1	0.5910	1.1537	1.4038
2	0.5672	1.1092	1.1053
3	0.5870	0.9835	1.0105
4	0.3478	0.5158	0.5089
Mean	0.523 ± 0.117	0.941 ± 0.292	1.007 ± 0.372

hCG group vs. control group *P* < 0.05; hCG/PD98059 group vs. control group *P* < 0.05; hCG/PD98059 group vs. hCG group *P* > 0.05.

**Table 7:** VEGF mRNA relative expression levels after hCG/PD98059 treating.

Sample no.	Control group	hCG group	hCG/PD98059
1	1	2.3703	1.6896
2	1	3.3180	1.4159
3	1	2.0062	0.8044
4	1	1.4885	0.6069
Mean	1	2.295 ± 0.771	1.1292 ± 0.5081

hCG group vs. control group *P* < 0.05; hCG/PD98059 group vs. control group *P* > 0.05; hCG/PD98059 group vs. hCG group *P* < 0.05.

**Table 8:** VEGF protein relative expression levels after hCG/PD98059 treating.

Sample no.	Control group	hCG group	hCG/PD98059
1	0.8204	1.5049	0.9178
2	0.6934	1.4837	0.6951
3	0.6025	1.0913	0.7351
4	0.4065	0.6094	0.3672
Mean	0.631 ± 0.174	1.172 ± 0.421	0.678 ± 0.229

hCG group vs. control group *P* < 0.05; hCG/PD98059 group vs. control group *P* > 0.05; hCG/PD98059 group vs. hCG group *P* < 0.05.

## Data Availability

The datasets used and/or analyzed during the current study are available from the corresponding author on reasonable request.

## References

[1] Rao C. V. (2001). Multiple novel roles of luteinizing hormone. *Fertility and Sterility*.

[2] Licht P., Von Wolff M., Berkholz A., Wildt L. (2003). Evidence for cycle-dependent expression of full-length human chorionic gonadotropin/luteinizing hormone receptor mRNA in human endometrium and decidua. *Fertility and Sterility*.

[3] Sacchi S., Sena P., Degli Esposti C., Lui J., La Marca A. (2018). Evidence for expression and functionality of FSH and LH/hCG receptors in human endometrium. *Journal of Assisted Reproduction and Genetics*.

[4] Makrigiannakis A., Vrekoussis T., Zoumakis E., Kalantaridou S. N., Jeschke U. (2017). The role of hcg in implantation: a mini-review of molecular and clinical evidence. *International Journal of Molecular Sciences*.

[5] Lessey B. A., Stanley R. G., Aplin J. (2002). *Uterine Factors in Implantantion. The Endometrium*.

[6] Tranguch S., Daikoku T., Guo Y., Wang H., Dey S. K. (2005). Molecular complexity in establishing uterine receptivity and implantation. *Cellular and Molecular Life Sciences*.

[7] Marsh C., Schumacher K., Nothnick W. B., Taylor R. N., Monard M., Litsenko O. I. (2018). Secretory phase and implantation. *Menstrual Cycle*.

[8] Mirsaeidi M., Gidfar S., Vu A., Schraufnagel D. (2016). Annexins family: insights into their functions and potential role in pathogenesis of sarcoidosis. *Journal of Translational Medicine*.

[9] Serban D., Leng J., Cheresh D. (2008). H-ras regulates angiogenesis and vascular permeability by activation of distinct downstream effectors. *Circulation Research*.

[10] Binder N. K., Evans J., Gardner D. K., Salamonsen L. A., Hannan N. J. (2014). Endometrial signals improve embryo outcome: functional role of vascular endothelial growth factor isoforms on embryo development and implantation in mice. *Human Reproduction*.

[11] Wang B., Ye T. M., Lee K. F. (2015). Annexin A2 acts as an adhesion molecule on the endometrial epithelium during implantation in mice. *PLoS One*.

[12] Lei Z. M., Toth P., Rao C. V., Pridham D. (1993). Novel coexpression of human chorionic gonadotropin (hCG)/human luteinizing hormone receptors and their ligand hCG in human fallopian tubes. *The Journal of Clinical Endocrinology & Metabolism*.

[13] Fields M. J., Shemesh M. (2004). Extragonadal luteinizing hormone receptors in the reproductive tract of domestic animals1. *Biology of Reproduction*.

[14] Yu W. J., Li J. (2009). *Expression of Annexin IV and StAR on Endometrial Tissue and its Regulation by hCG*.

[15] Li Y.-X., Guo X., Gulappa T., Menon B., Menon K. M. J. (2019). SREBP plays a regulatory role in LH/hCG receptor mRNA expression in human granulosa-lutein cells. *The Journal of Clinical Endocrinology & Metabolism*.

[16] Arroyo A., Kim B., Yeh J. (2020). Luteinizing hormone action in human oocyte maturation and quality: signaling pathways, regulation, and clinical impact. *Reproductive Sciences*.

[17] Robker R. L., Hennebold J. D., Russell D. L. (2018). Coordination of ovulation and oocyte maturation: a good egg at the right time. *Endocrinology*.

[18] Sivakumar R., Balasubramanian K. (2005). Effects of gamma radiation on luteinizing hormone (LH) receptor expression, signal transduction and steroidogenesis in cultured rat leydig cells. *International Journal of Radiation Biology*.

[19] Wang W., Qiao Y., Li Z. (2018). New insights into modes of GPCR activation. *Trends in Pharmacological Sciences*.

[20] Kang S. K., Tai C.-J., Nathwani P. S., Choi K.-C., Leung P. C. K. (2001). Stimulation of mitogen-activated protein kinase by gonadotropin-releasing hormone in human granulosa-luteal cells∗∗ this work was supported grants from the medical research council of Canada. *Endocrinology*.

[21] Yang M., Wang X., Wang L., Wang X., Li J., Yang Z. (2017). IL-1*α* up-regulates IL-6 expression in bovine granulosa cells via MAPKs and NF-*κ*B signaling pathways. *Cellular Physiology and Biochemistry*.

[22] Dewi D. A., Abayasekara D. R. E., Wheeler-Jones C. P. D. (2002). Requirement for ERK1/2 activation in the regulation of progesterone production in human granulosa-lutein cells is stimulus specific. *Endocrinology*.

[23] Riccetti L., De Pascali F., Gilioli L. (2017). Human LH and hCG stimulate differently the early signalling pathways but result in equal testosterone synthesis in mouse leydig cells in vitro. *Reprod Biol Endocrinol*.

[24] Gotoh M., Takamoto Y., Kurosaka K. (2005). Annexins I and IV inhibit staphylococcus aureus attachment to human macrophages. *Immunology Letters*.

[25] Matteo R., Moravec C. S. (2000). Immunolocalization of annexins IV, V and VI in the failing and non-failing human heart. *Cardiovascular Research*.

[26] Mirkin S., Arslan M., Churikov D. (2005). In search of candidate genes critically expressed in the human endometrium during the window of implantation. *Human Reproduction*.

[27] Ponnampalam A. P., Rogers P. A. W. (2006). Cyclic changes and hormonal regulation of annexin IV mRNA and protein in human endometrium. *MHR: Basic Science of Reproductive Medicine*.

[28] Yu W. J., Li J., Tan Z., Liu Q. (2008). Changes of annexin during the menstrual cycle on endometrial tissue. *Chinese Journal of Practical Gynecology and Obstetrics*.

[29] Bukowska D., Kempisty B., Jackowska M. (2011). Analysis of integrins and vascular endothelial growth factor isoforms mRNA expression in the canine uterus during perimplantation period. *Polish Journal of Veterinary Sciences*.

[30] Valdés G., Corthorn J. (2011). Review: the angiogenic and vasodilatory utero-placental network. *Placenta*.

[31] Souza C., Ocarino N., Silva J. (2011). Administration of thyroxine affects the morphometric parameters and VEGF expression in the uterus and placenta and the uterine vascularization but does not affect reproductive parameters in gilts during early gestation. *Reproduction in Domestic Animals*.

[32] Hosper N. A., Eggink A. J., Roelofs L. A. J. (2010). Intra-uterine tissue engineering of full-thickness skin defects in a fetal sheep model. *Biomaterials*.

[33] Licht P., Russu V., Lehmeyer S., Wildt L. (2001). Molecular aspects of direct LH/hCG effects on human endometrium--lessons from intrauterine microdialysis in the human female in vivo. *Reproductive Biology*.

[34] Licht P., Russu V., Wildt L. (2001). On the role of human chorionic gonadotropin (hCG) in the embryo-endometrial microenvironment: implications for differentiation and implantation. *Seminars in Reproductive Medicine*.

[35] Berndt S., Perrier d’Hauterive S., Blacher S. (2006). Angiogenic activity of human chorionic gonadotropin through LH receptor activation on endothelial and epithelial cells of the endometrium. *The FASEB Journal*.

[36] Fogle R. H., Li A., Paulson R. J. (2010). Modulation of HOXA10 and other markers of endometrial receptivity by age and human chorionic gonadotropin in an endometrial explant model. *Fertility and Sterility*.

[37] Paiva P., Hannan N. J., Hincks C. (2011). Human chorionic gonadotrophin regulates FGF2 and other cytokines produced by human endometrial epithelial cells, providing a mechanism for enhancing endometrial receptivity. *Human Reproduction*.

